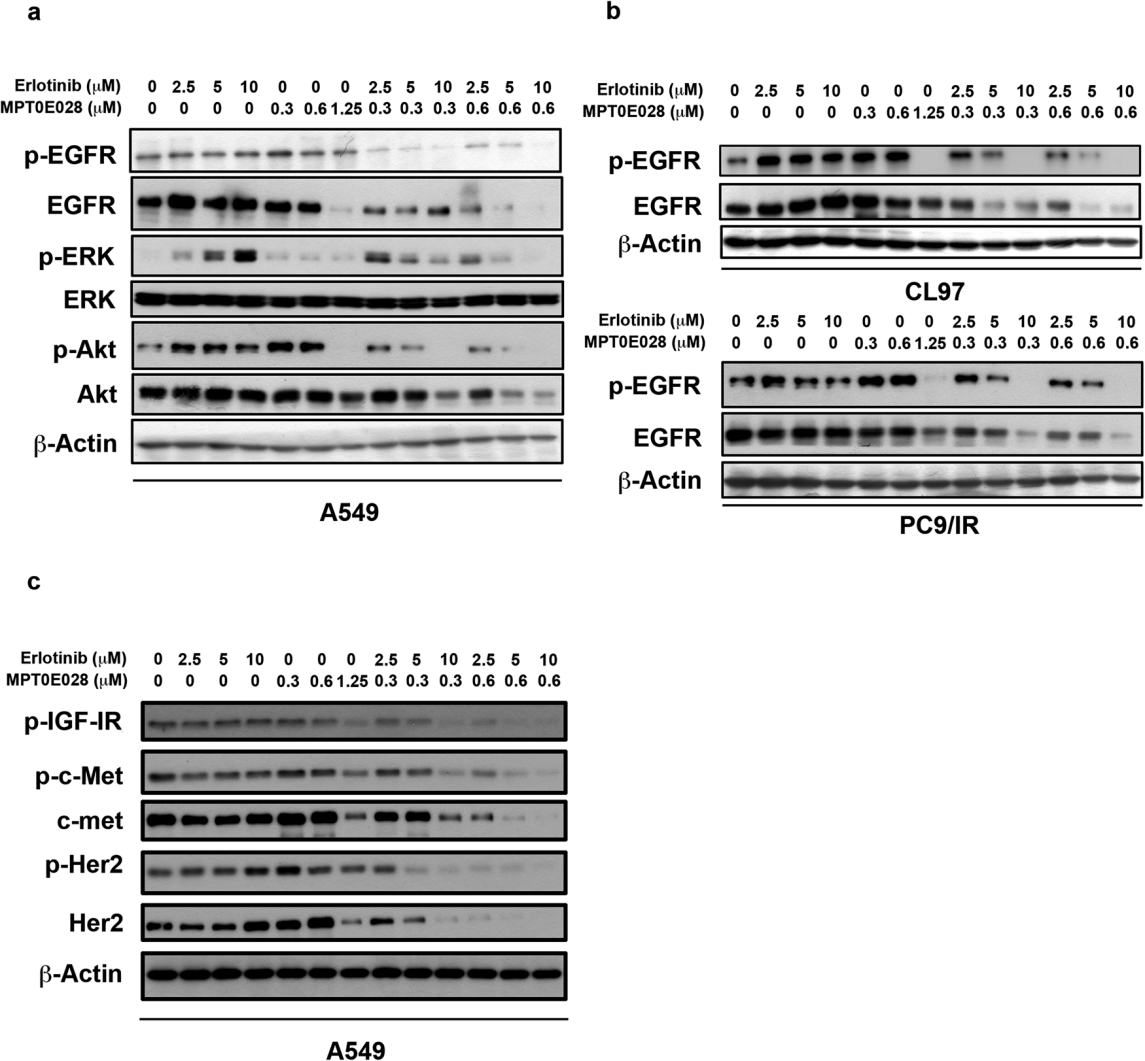# Correction: The HDAC inhibitor, MPT0E028, enhances erlotinib induced cell death in EGFR-TKI-resistant NSCLC cells

**DOI:** 10.1038/s41419-024-06794-4

**Published:** 2024-07-09

**Authors:** M. -C Chen, C. -H Chen, J. -C Wang, A. -C Tsai, J. -P Liou, S. -L Pan, C. -M Teng

**Affiliations:** 1 Pharmacological Institute, College of Medicine, National Taiwan University, Taipei, Taiwan; 2 The PhD Program for Cancer Biology and Drug Discovery, College of Medical Science and Technology, Taipei Medical University, Taipei, Taiwan; 3 School of Pharmacy, College of Pharmacy, Taipei Medical University, Taipei, Taiwan

Correction to: *Cell Death and Disease* 10.1038/cddis.2013.330, published online 19 September 2013

In Figure 4e (Ac-tubulin) and Figure 5b (p-EGFR), the western blots were inadvertently misplaced during final figure preparation. The results have been confirmed by three replicates and the figures have been corrected accordingly. These corrections do not affect the results and conclusions of this study. We deeply apologize for any confusion or inconvenience that this may have caused. The original article has been corrected.

The original blots of three replicates for the correction of 4e:
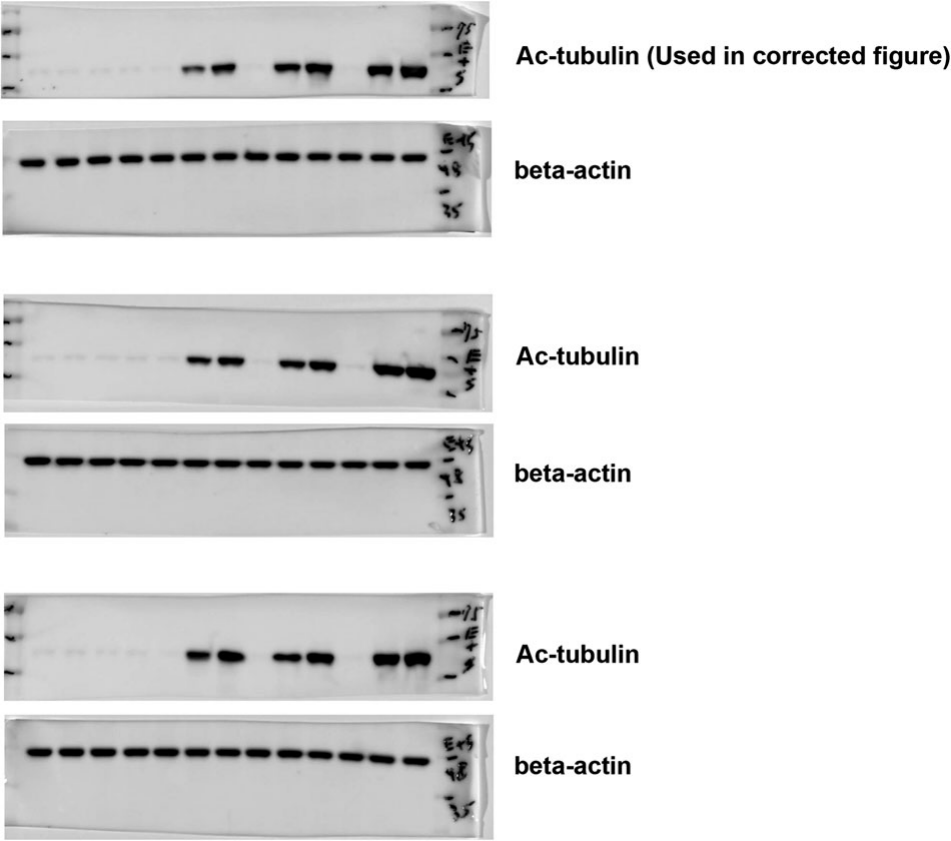


Corrected figure 4:
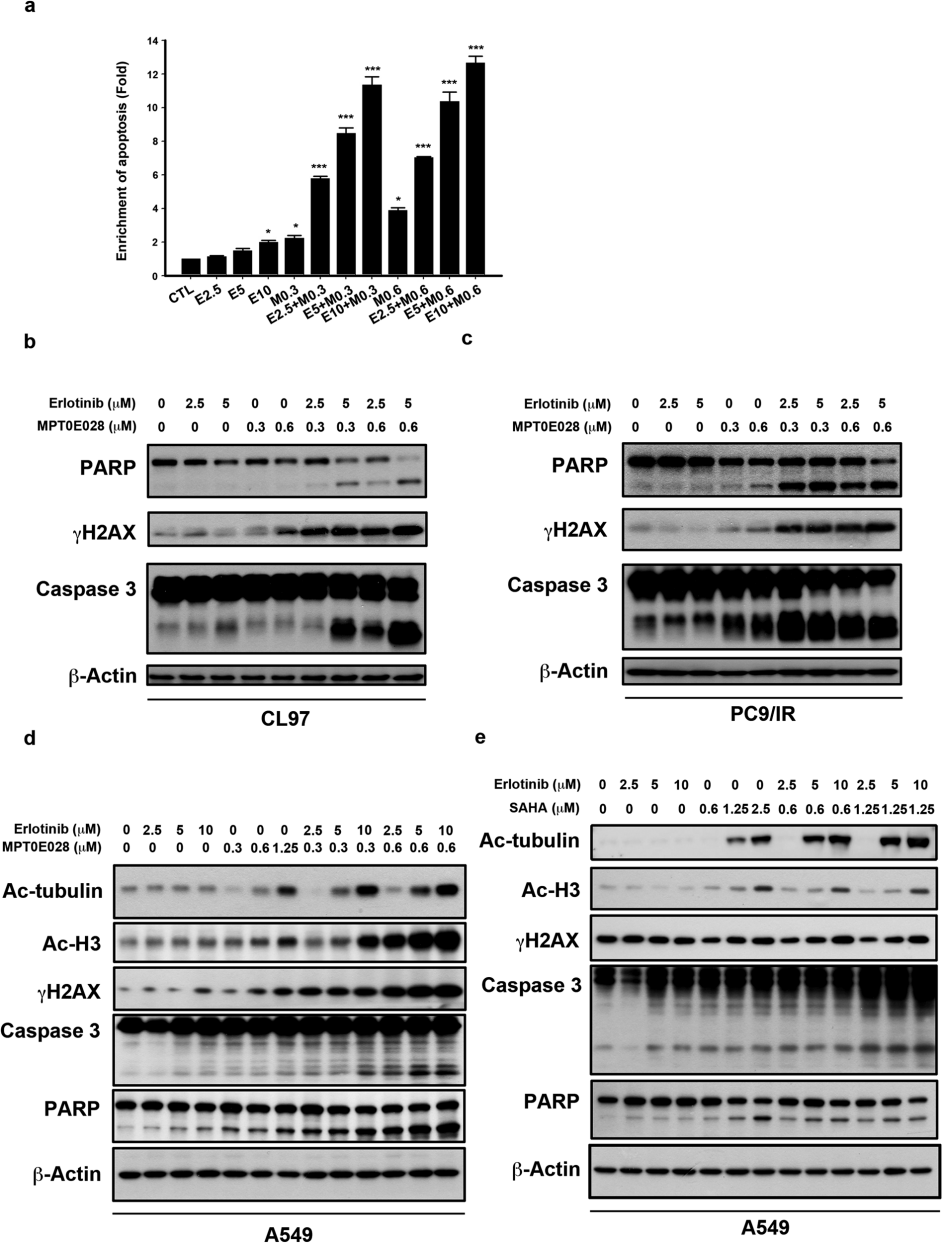


The original blots of three replicates for the correction of 5b:
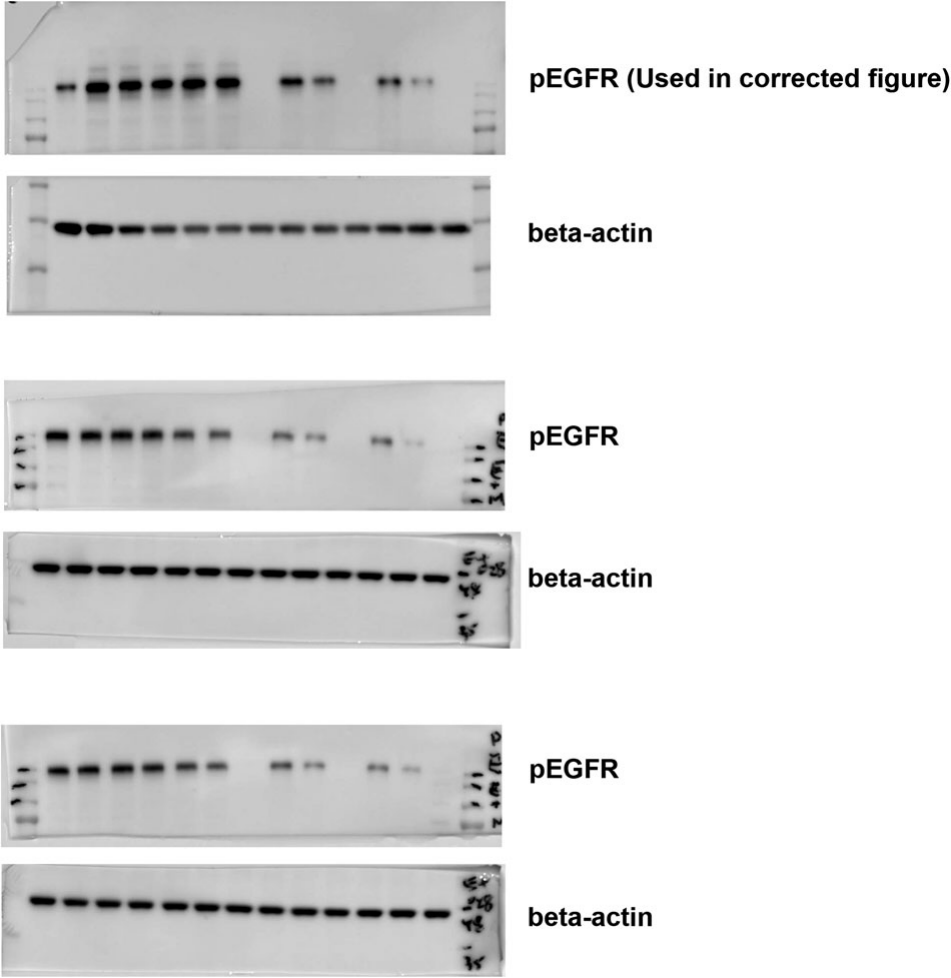


Corrected figure 5: